# Effects of Foliar Application of Paclobutrazol on Grain Yield, Aroma, and Canopy Radiation Use Efficiency of Aromatic Rice

**DOI:** 10.3390/biology14111562

**Published:** 2025-11-07

**Authors:** Fengqin Hu, Jian Lu, Laiyuan Zhai, Xianjin Qiu, Bin Du, Jianlong Xu

**Affiliations:** 1 College of Agriculture, Yangtze University, Jingzhou 434022, China; 2 State Key Laboratory of Crop Gene Resources and Breeding/Institute of Crop Sciences, Chinese Academy of Agricultural Sciences, Beijing 100081, China; 3 Chongqing Academy of Agricultural Sciences, Chongqing 400000, China; 4 Guangdong Laboratory of Lingnan Modern Agriculture, Genome Analysis Laboratory of the Ministry of Agriculture, Agricultural Genomics Institute at Shenzhen, Chinese Academy of Agricultural Sciences, Shenzhen 518120, China; 5 National Nanfan Research Institute (Sanya), Chinese Academy of Agricultural Sciences, Sanya 572024, China

**Keywords:** 2-acetyl-1-pyrroline, aromatic rice, net photosynthetic rate, paclobutrazol, radiation use efficiency

## Abstract

**Simple Summary:**

The effects of paclobutrazol application on grain yield and aroma in aromatic rice remain underexplored. This study demonstrated that foliar spraying of paclobutrazol at an optimal concentration (150 mg L^−1^) increased canopy radiation use efficiency, thus enhancing both grain yield and 2-acetyl-1-pyrroline content. Additionally, paclobutrazol treatment improved photosynthetic performance and promoted the synthesis of key aroma precursors. These findings provide a novel feasible chemical regulation strategy for achieving high-yield aromatic rice production with superior aroma quality.

**Abstract:**

Paclobutrazol (PBZ) is extensively used to modulate plant architecture in rice. However, its comprehensive effects on grain yield and aroma in aromatic rice have not been thoroughly investigated. This study used the local aromatic rice cultivars (Meixiangzhan 2 and Xiangyaxiangzhan) as experimental materials to evaluate the impacts of foliar-applied PBZ at three concentrations (0 (CK), 150 (T1), and 300 (T2) mg L^−1^) on grain yield, photosynthetic characteristics, fragrance formation, and radiation use efficiency (RUE). Field experiments revealed that T1 significantly reduced the leaf area index (LAI) by 10.12% and intercepted photosynthetically active radiation (IPAR) by 10.74%, meanwhile significantly increasing SPAD values by 12.94% and net photosynthetic rate (Pn) by 9.95%, leading to improved RUE up to 25.21%. These changes contributed to a larger number of grains per panicle and increased 1000-grain weight, ultimately enhancing grain yield. In contrast, T2 resulted in a sharp reduction by 24.84% in IPAR and a significant decline in Pn by 10.07% during the late grain-filling stage, thus limiting the supply of photosynthetic assimilates, eventually reducing grain yield. PBZ application also significantly elevated 2-acetyl-1-pyrroline (2-AP) content by 28.74% under T1 and 17.51% under T2, compared to the control. The increase in 2-AP was mainly associated with elevated levels of key precursors, including proline, Δ1-pyrroline-5-carboxylic acid, and Δ1-pyrroline. In spite of differences in traits between cultivars, the traits responded to PBZ in the same pattern. These results indicate that foliar application of PBZ at 150 mg L^−1^ can effectively improve both yield and aroma of aromatic rice, offering a promising cultivation strategy for high-quality aromatic rice production.

## 1. Introduction

Aromatic rice is highly favored by consumers for its distinctive fragrance and superior taste [1]. Despite being priced 30–50% higher than conventional high-quality rice, its global consumption continues to grow annually [2,3]. However, the yield potential of aromatic rice is relatively lower than that of non-aromatic rice, due to its weaker adaptability to environmental changes and greater susceptibility to diseases during the growth process [4]. Therefore, enhancing grain yield while improving aroma content has become a key research objective.

2-Acetyl-1-pyrroline (2-AP) is the primary volatile compound responsible for the characteristic aroma of aromatic rice [5,6]. Its biosynthesis is regulated by key genes such as *BADH2*, and also influenced by external agronomic factors such as temperature, light, micronutrient availability, and cultivation techniques [7,8,9,10,11]. Several previous studies have shown that appropriate concentrations of plant growth regulators can concurrently increase grain yield and 2-AP content, offering a promising chemical strategy for “aroma-enhancing cultivation” [12,13,14]. Paclobutrazol (PBZ), a triazole-type plant growth regulator, significantly influences plant growth and development by inhibiting sterol and gibberellin biosynthesis, thereby modulating photosynthetic efficiency and endogenous hormone levels [15,16]. Previous studies have demonstrated that PBZ application can reduce plant height, increase stem thickness and chlorophyll content, and ultimately raise grain yield by 20–30% [17]. Xing et al. [12] found that foliar spraying of 100–120 mg L^−1^ PBZ at the heading stage increased grain 2-AP content by 18.80%. Further research indicated that seed priming with PBZ also enhanced pigment content and net photosynthetic rate in aromatic rice seedlings, establishing a physiological basis for improved yield and quality [18]. Moreover, PBZ promotes the accumulation of free proline in plants [19], which serves not only as an osmoprotectant enhancing abiotic stress tolerance but also as a key precursor for 2-AP synthesis [20,21].

Although the roles of PBZ in dwarfing plants, improving lodging resistance, and increasing yield have been well documented [17,19,22], its influence on canopy architecture and subsequent effects on intercepted photosynthetically active radiation (IPAR), and radiation use efficiency (RUE), as well as its ultimate effect on grain yield and aroma of aromatic rice, remain poorly understood. To address this gap, we selected two prominent aromatic rice cultivars widely cultivated in South China, Meixiangzhan 2 (MXZ2) and Xiangyaxiangzhan (XYXZ), due to their superior aroma and taste. PBZ was applied as a single foliar spray at the panicle initiation stage at three concentrations: 0 mg L^−1^ (CK), 150 mg L^−1^ (T1), and 300 mg L^−1^ (T2). By systematically assessing leaf photosynthetic performance, IPAR, and RUE under PBZ treatments, this study aimed to clarify the relationship between canopy light utilization efficiency and yield or aroma accumulation. The results showed that foliar application of PBZ at the optimal concentration of 150 mg L^−1^ effectively increased both grain yield and 2-AP content by improving canopy light utilization and photosynthetic efficiency. Our findings will provide a theoretical basis and practical references for high-quality and efficient aromatic rice cultivation.

## 2. Materials and Methods

### 2.1. Plant Materials, Growth Conditions, and Experimental Design

In this study, two indica aromatic rice cultivars, “Meixiangzhan 2 (MXZ2) and Xiangyaxiangzhan (XYXZ)”, were used as experimental materials. Field experiments were conducted from early July to late October in 2022 and 2023 at Jiangmen City (22°17.8′ N, 113°03.4′ E), Guangdong Province, China. The experimental soil was sandy loam, and the physical and chemical properties of the 0–20 cm soil layer are as follows: total nitrogen (N) 1.49 g kg^−1^, total phosphorus (P) 0.63 g kg^−1^, total potassium (K) 16.3 g kg^−1^, available P 22.4 mg kg^−1^, available K 86 mg kg^−1^, organic C 15.7 g kg^−1^, and pH 5.5.

The experiment followed a split-plot design, with the main plots arranged in three PBZ concentration levels: CK (0 mg L^−1^), T1 (150 mg L^−1^), and T2 (300 mg L^−1^). A foliar spray of PBZ was administered at the panicle initiation stage. Subplots within each main plot were planted with different rice cultivars. All treatments were replicated three times in plots of 40 m^2^. Transplantation was carried out at the three-leaf and one-heart stage, using a hill spacing of 20 cm × 30 cm and two seedlings per hill. A total of 900 kg ha^−1^ of compound fertilizer (N-P_2_O_5_-K_2_O=15-15-15, Huaqiang Chemical Group Co., Ltd., Yichang, Hubei, China) was applied throughout the growth period, including 450 kg ha^−1^ as base fertilizer, 270 kg ha^−1^ as tillering fertilizer, and 180 kg ha^−1^ as top dressing at the booting stage. Other cultivation measures were carried out in accordance with the local high-yield cultivation standards. Field management according to the production field routine management.

### 2.2. Measurements

#### 2.2.1. Above-Ground Dry Matter Accumulation

A total of 15 plants were taken from each plot by the five-point sampling method at full heading stage (FH), 14 days after heading (FH14), and at maturity (MA), respectively. The roots were removed, and the above-ground parts were retained. The samples were dried at 80 °C until constant weight. After cooling, the samples were weighed with an electronic balance (accurate to 0.001 g).

#### 2.2.2. Yield Evaluation

After the rice matured, “the five-point sampling method” was uniformly adopted for sampling. In each plot, 5 m^2^ were selected for measurement of grain yield (GY, t ha^−1^). After manual threshing, a small number of grain samples were randomly taken, and the moisture content was measured with a grain moisture rapid tester (HED-L80). According to a 13.5% standard water content, the dry weight was converted to calculate the actual yield of the plot. Meanwhile, 30 rice plants with consistent growth were selected to investigate the effective panicle number (EPN), grain number per panicle (GNP), and seed setting rate (SSR, %). One thousand grains of air-dried rice were randomly selected and weighed, and the average 1000-grain weight (TGW, g) was obtained by repeating 3 times.

#### 2.2.3. Photosynthetic Related Parameters

During the FH, FH14, and MA growth stages, thirty flag leaves exhibiting uniform growth were selected from each plot between 9:00 and 11:00 a.m. on sunny days. The net photosynthetic rate (Pn, µmol m^−2^ s^−1^) was measured with a portable LI-6800XT photosynthesis system (LI-COR Biosciences, Lincoln, NE, USA), and the SPAD value was determined using a SPAD-502 chlorophyll meter (Konica Minolta Sensing Europe B.V., Nieuwegein, The Netherlands). Meanwhile, five plants of comparable growth were selected from each plot, and the leaf area index (LAI) was determined using a millimeter-precision steel ruler and a conversion coefficient of 0.75 [23].

#### 2.2.4. Grain 2-Acetyl-1-Pyrroline Content

After the rice matured, fresh grains from the main stem were harvested, immediately frozen in liquid nitrogen, and stored at −20 °C. The 2-acetyl-1-pyrroline (2-AP, µg kg^−1^) content was determined by synchronized distillation and extraction method combined with GCMS-QP 2010 Plus (Shimadzu Corporation, Kyoto, Japan) as described by Mo et al. [11]. Each measurement was repeated three times.

#### 2.2.5. Grains Δ1-Pyrroline-5-Carboxylic Acid, Proline, and Δ1-Pyrroline Contents

Proline (µg g^−1^) and Δ1-pyrroline-5-carboxylic acid (P5C, µmol g^−1^) contents in aromatic rice were quantified following the methodology described by Li et al. [5], while Δ1-pyrroline content (mmol g^−1^) was estimated in accordance with the procedures outlined by Bao et al. [24]. Each measurement was repeated three times.

#### 2.2.6. Intercepted Radiation and Radiation Use Efficiency

At the FH, FH14, and MA stages, the light interception rate of the canopy was measured between 11:00 and 13:00 (sunny days) using the SunScan Canopy Analysis System (Delta-T Devices Ltd., Burwell, Cambridge, UK). Canopy light intensity was measured according to Lu et al. [25]. A light sensor probe was placed between rows just above the water surface in each plot, with four replicates per measurement both within and between rows. The canopy light interception rate was derived as follows: 100 × (incident light intensity–canopy interior light intensity)/incident light intensity. The intercepted photosynthetically active radiation (IPAR, MJ m^−2^) per growth stage was estimated as follows: 1/2 × (initial interception rate + final interception rate) × cumulative stage radiation. Radiation use efficiency (RUE, g MJ^−1^) was calculated as the total above-ground dry matter accumulation divided by the total IPAR for each stage. Daily solar radiation, as well as minimum and maximum temperatures, were automatically recorded using a Vantage Pro2 weather station (Davis Instruments, Hayward, CA, USA).

### 2.3. Data Processing and Analysis

Data entry and collation were performed using Microsoft Excel 2016. Statistical analyses were conducted with R 4.5.1, and analysis of variance (ANOVA) was employed to test for significance. The results were visualized using Origin 2023.

## 3. Results

### 3.1. Above-Ground Biomass

The response of above-ground biomass to PBZ exhibited varying trends at different growth stages (Table 1). Analysis of variance (ANOVA) revealed no significant differences in above-ground biomass between years, cultivars, treatments, or their interactions at the FH. At the FH14 stage, however, significant differences in above-ground biomass were observed across years, treatments, and their interaction. Under T1 and T2, the average above-ground biomass of the two cultivars increased by 10.78% and 6.06% in 2023, and by 5.90% and 8.40% in 2024, respectively, compared to the CK. At the MA stage, significant differences in above-ground biomass were detected only for treatments and the interaction between year and cultivar. The two-year average above-ground biomass under T1 and T2 was 1312.94 g m^−2^ and 1198.19 g m^−2^, respectively, compared with 1244.26 g m^−2^ under CK. This indicates that T1 had extremely significantly higher above-ground biomass than both CK and T2, while T2 was also significantly less than CK.

### 3.2. Yield and Yield Composition

**Figure 1:**
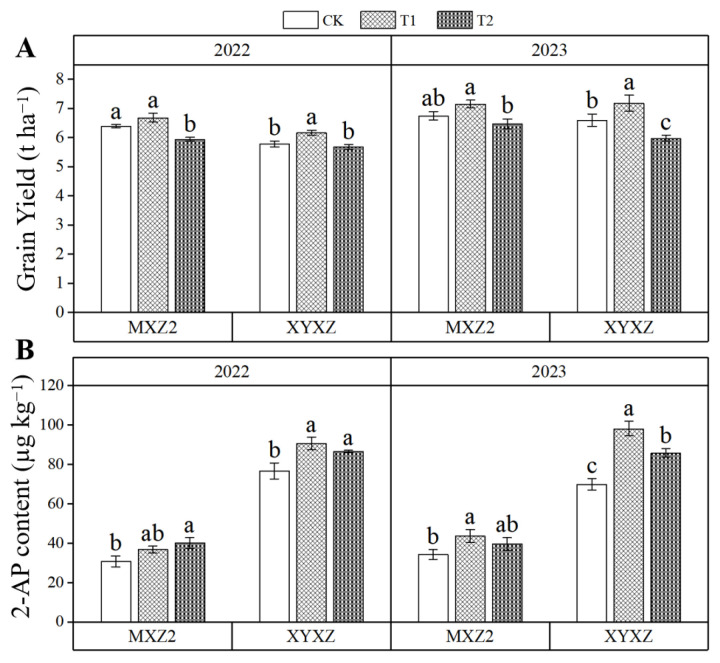
Effect of different paclobutrazol concentrations on grain yield and 2-AP content at maturity stage. Note: 2-AP, 2-acetyl-1-pyrroline; CK, water control; T1, 150 mg L^−1^ PBZ; T2, 300 mg L^−1^ PBZ; MXZ2, Meixiangzhan 2; XYXZ, Xiangyaxiangzhan. (**A**) Effects of different paclobutrazol treatments on grain yield of MXZ2 and XYXZ in 2022 and 2023. (**B**) Effects of different paclobutrazol treatments on grain 2-AP content of MXZ2 and XYXZ in 2022 and 2023. Different lowercase letters indicate significant differences between treatments in the same year at *p* < 0.05.

Differential grain yield responses to PBZ were observed between the cultivars (Figure 1A). T1 significantly enhanced yield in XYXZ relative to both T2 and CK. However, MXZ2 showed a more moderate response, with the yield following the order of T1 > CK > T2 (not statistically significant between CK and T1). These results showed that T1 had a promoting effect on yield, with a more pronounced promoting effect in XYXZ than in MXZ2. Therefore, an appropriate concentration of PBZ promoted yield, but excessively high concentrations of PBZ resulted in yield reduction.

There were no significant differences in yield components between years. Therefore, we combined the data from the two years for analysis (Table 2). EPN and GNP exhibited notable variations among cultivars, with MXZ2 having EPN and GNP average values of 290.33 and 140.15, markedly significantly higher than 261.46 and 126.87 of XYXZ, respectively. The paclobutrazol treatment had no significant impact on EPN but exerted a significant (*p* < 0.01) effect on GNP with an average of 141.07 of T1 and significantly (*p* < 0.01) higher than 135.19 of CK and 124.26 of T2, respectively. The paclobutrazol treatment had no significant effects on SSR and TGW among years and cultivars except for treatments. The treatments consistently showed that the T1 had an average of 83.38% for SSR and 19.98 g for TGW, significantly higher than those of T2 (75.55% for SSR and 17.50 g for TGW) and CK (75.48% for SSR and 18.77 g for TGW), respectively.

### 3.3. Photosynthetic Characteristics

**Figure 2:**
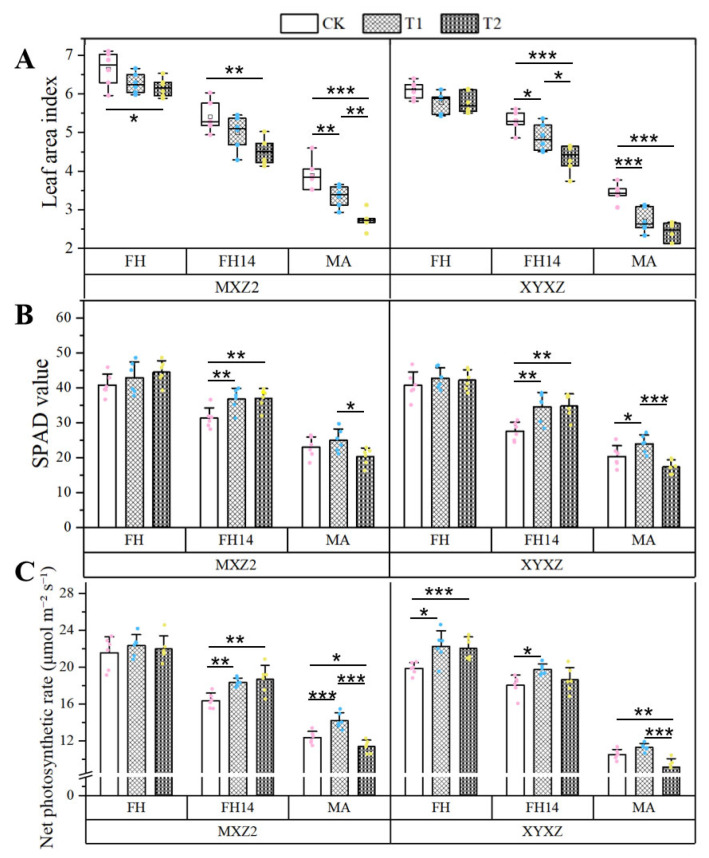
The effects of foliar application of paclobutrazol on photosynthetic characteristics of aromatic rice. Note: CK, water control; T1, 150 mg L^−^1 PBZ; T2, 300 mg L^−1^ PBZ; FH, full heading stage; FH14, 14 days after heading; MA, maturity stage. MXZ2, Meixiangzhan 2; XYXZ, Xiangyaxiangzhan. (**A**) Leaf area index under different concentrations of paclobutrazol; (**B**) SPAD value under different concentrations of paclobutrazol; (**C**) Net photosynthetic rate under different concentrations of paclobutrazol. Asterisks represent a significant difference between treatments determined by Student’s *t*-test. *, *p* < 0.05; **, *p* < 0.01; ***, *p* < 0.001.

As the rice growth stage progressed, the leaf area index (LAI), SPAD value, and net photosynthetic rate (Pn) all exhibited a declining trend (Figure 2). Compared with CK, foliar application of PBZ in both T1 and T2 significantly reduced LAI by 9.03% and 15.66% for the two cultivars, respectively. Additionally, both the SPAD value and Pn exhibited a consistent response trend to PBZ across different growth stages. At the FH, both T1 and T2 exerted a promoted effect on SPAD value and Pn, and this trend was more significant in the FH14 stage. However, at the MA stage, although the SPAD value and Pn under T1 remained significantly higher than those in CK, the T2 treatment exhibited a significant decline compared to CK, with Pn decreasing to a significantly (*p* < 0.01) different level.

### 3.4. Canopy Light Energy Resource Utilization

As shown in Table 3, no significant differences in IPAR or RUE were detected across years, with the exception of RUE at the FH14 stage. In contrast, significant differences were observed among treatments for both parameters, except for RUE at the MA stage. Compared with CK, T1 and T2 significantly reduced IPAR by 10.46% and 23.21% at the FH, and by 8.36% and 23.43% at the FH14 stage, respectively. Due to the significant genotypic variation in IPAR at the MA stage, treatment effects were analyzed separately for each cultivar. For MXZ2, IPAR under T2 was significantly lower than that under CK and T1, with reductions of 22.54% and 16.93%, respectively, while no significant difference was observed between CK and T1. For XYXZ, T2 resulted in an extremely significant reduction in IPAR compared to CK and T1, by 33.76% and 16.43%, respectively; moreover, T1 also led to a significant decrease of 16.43% relative to CK.

Cultivar-dependent responses of RUE to PBZ were observed. At the FH, RUE under T2 was significantly higher than that under CK and T1 in both cultivars, increasing by 31.74% and 20.33% in MXZ2, and by 41.38% and 18.47% in XYXZ, respectively. At the MA stage, no significant differences in RUE were detected among treatments for MXZ2, whereas for XYXZ, T1 was significantly higher than both CK and T2, with increases of 28.89% and 25.70%, respectively. At the FH14 stage, RUE varied significantly across years and treatments. Specifically, in 2022, both T1 and T2 treatments resulted in markedly higher RUE than CK, with increases of 59.62% and 79.91%, respectively. In 2023, however, only T2 showed a significant improvement over CK, with an increase of 36.76%. Interactions among most factors significantly influenced both IPAR and RUE, suggesting that the expression of these traits is more complex than that of grain yield and above-ground biomass. Overall, both T1 and T2 reduced IPAR. but T2 exhibited a stronger ability to reduce IPAR than T1. T1 increased RUE throughout the grain-filling stage, while T2 resulted in a first increase (early grain-filling stage) and then a decrease (late grain-filling stage) in RUE.

### 3.5. Grain 2-Acetyl-1-Pyrroline, Proline, P5C, and Δ1-Pyrroline Contents

Over two consecutive seasons, PBZ application significantly increased grain 2-acetyl-1-pyrroline (2-AP) content in both MXZ2 and XYXZ (Figure 1B). In MXZ2, the T2 led to a significant increase of 30.14% compared with CK in 2022, whereas the T1, despite a 19.31% increase, did not reach statistical significance. In 2023, T1 resulted in a significant 27.36% rise relative to CK, while T2 showed a 15.57% increase that was not statistically significant. For XYXZ, both T1 and T2 significantly enhanced 2-AP content compared to CK across both years, with average increases of 29.29% and 17.81%, respectively. Overall, both T1 and T2 markedly elevated 2-AP levels relative to the control, with T1 exhibiting a stronger enhancement effect, thereby contributing to a more pronounced fragrance in the two aromatic cultivars.

**Figure 3:**
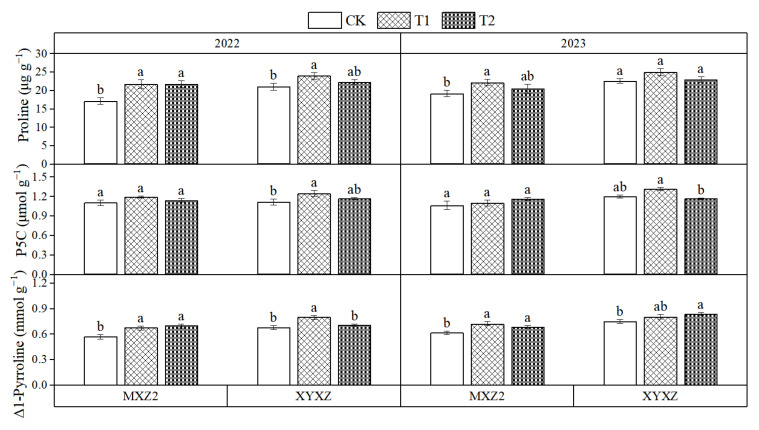
Effects of different paclobutrazol treatments on Proline, P5C, and Δ1-Pyrroline Contents. Note: P5C, Δ1-pyrroline-5-carboxylic acid; CK, water control; T1, 150 mg L^−1^ PBZ; T2, 300 mg L^−1^ PBZ; MXZ2, Meixiangzhan 2; XYXZ, Xiangyaxiangzhan. Different lowercase letters indicate significant differences between treatments in the same year at the level of *p* < 0.05.

Compared to CK, both T1 and T2 elevated the contents of proline, P5C, and Δ1-pyrroline in the grains of MXZ2 and XYXZ over the two-year study (Figure 3). The T1, in particular, consistently outperformed CK across both cultivars and years, increasing proline content by approximately 21.26% in MXZ2 and 12.23% in XYXZ, P5C content by about 5.67% and 10.49%, and Δ1-pyrroline content by around 17.70% and 12.15%, respectively. The above results showed that foliar PBZ concurrently raised 2-AP and its precursors, with T1 displaying a larger increment than T2.

### 3.6. Correlation Coefficient Analysis

**Figure 4:**
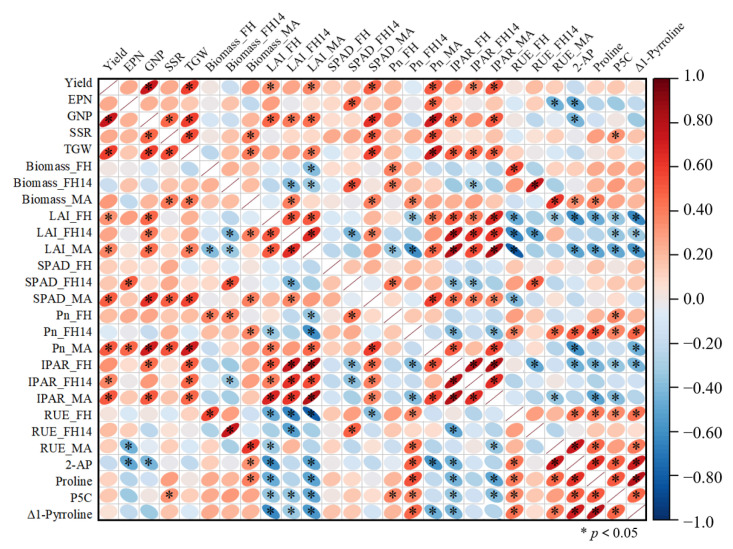
Correlation between parameters. Notes: EPN, effective panicles number; GNP, grain number per panicle; SSR, seed setting rate; TGW, 1000-grain weight; LAI, leaf area index; Pn, net photosynthetic rate; IPAR, intercepted photosynthetically active radiation; RUE, radiation use efficiency; 2-AP, 2-acetyl-1-pyrroline; P5C, Δ1-pyrroline-5-carboxylic acid; FH, full heading stage; FH14, 14 days after heading; MA, maturity stage.

Figure 4 illustrates the correlations among various parameters. Yield showed significant positive correlations with GNP, TGW, LAI at both FH and MA stages, SPAD and Pn at the MA stage, as well as IPAR at FH14 and MA stages. IPAR was strongly and positively correlated with LAI across all growth stages. Similarly, RUE was significantly positively related to above-ground biomass. Additionally, 2-AP content was significantly associated with biomass at the MA stage, Pn at the FH14 stage, and RUE at both the FH and MA stages, and precursors for 2-AP synthesis at the MA stage.

### 3.7. Interaction Analysis of 2-AP Content with IPAR and RUE

**Figure 5:**
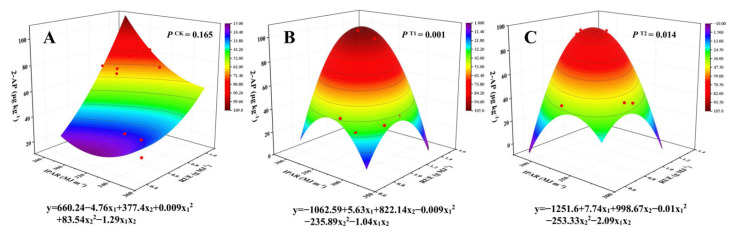
The interaction models of 2-AP content with IPAR and RUE. Note: CK, water control; T1, 150 mg L^−1^ PBZ; T2, 300 mg L^−1^ PBZ; 2-AP, 2-acetyl-1-pyrroline; IPAR, intercepted photosynthetically active radiation; RUE, radiation use efficiency. Figures (**A**–**C**) present the fitted surface models of 2-AP content in mature grains under CK, T1, and T2 with different levels of IPAR and RUE.

We found a significant relationship between 2-AP and RUE in the correlation analysis. To elucidate the relationship between 2-AP content and light energy utilization in aromatic rice under PBZ influence, several three-dimensional interaction models were established to characterize the interplay among 2-AP, IPAR, and RUE (Figure 5). The model was not statistically significant under the CK condition (*P*^CK^ = 0.165), but showed significant effects under both T1 and T2 (*P*^T1^ = 0.001 and *P*^T2^ = 0.014, respectively). The response of 2-AP content to IPAR and RUE followed a unimodal trend, initially increasing and then decreasing with rising IPAR or RUE. According to the model, relatively higher 2-AP content was achieved when both IPAR and RUE were maintained at intermediate levels.

## 4. Discussion

As a plant growth regulator, PBZ has been widely used in rice, corn, wheat, and other major crops [22]. However, the impact of PBZ on crop yield is concentration-dependent, with low concentrations of PBZ promoting yield and high concentrations inhibiting it [16,18,26,27]. The results of our study also support this conclusion. We found that foliar application of PBZ at 150 mg L^−1^ (T1) increased yield by raising GNP and TGW (Table 2; Figure 4). This might be because the higher SPAD value and net photosynthetic rate (Pn) under T1 lead to the accumulation and transport of photosynthetic products, thereby increasing the above-ground biomass [28]. The SPAD value is an important indicator for measuring chlorophyll content. PBZ application has been reported to promote cytokinin synthesis, thereby enhancing chloroplast development and chlorophyll biosynthesis, and maintaining high photosynthetic efficiency [29]. Moreover, Nouriyani et al. [30] have found that PBZ extends the duration of photosynthesis in the flag leaves by enhancing the contents of chlorophyll a, chlorophyll b, and total chlorophyll. Moreover, the application of PBZ has also been found to maintain the stability of chloroplast structure [31]. However, under the treatment of PBZ at 300 mg L^−1^ (T2), the SPAD value and Pn during the mature (MA) stage showed a downward trend compared to CK and T1 (Figure 2B). This downward trend may be due to the accelerated leaf senescence induced by the high PBZ concentration, which reduced chlorophyll content and photosynthetic efficiency [32]. During the later stage of grain filling, reduced photosynthetic efficiency led to an insufficient supply of nutrients, which in turn affected the filling degree of the grains, decreased TGW, and ultimately resulted in a reduction in yield [33].

Furthermore, we also observed that the application of PBZ at two concentrations reduced the LAI, with the T2 showing a more pronounced reduction (Figure 2A). LAI is closely related to IPAR. A decrease in LAI can lead to light leakage within the canopy, resulting in reduced light interception by the canopy and consequently a decline in IPAR [32,34]. IPAR reflects the light interception capability of the canopy, while RUE indicates the efficiency with which plants convert intercepted photosynthetically active radiation into dry matter [35,36,37]. In this study, compared to the control without PBZ (CK), the T1 significantly reduced IPAR in the canopy after heading and increased RUE (Table 3). This indicates that an appropriate concentration of PBZ can enhance the rice canopy’s ability to intercept and convert photosynthetically active radiation, ensuring sufficient accumulation of photosynthetic products during the grain-filling period, thereby increasing yield. However, the T2 exhibited a more substantial decline in IPAR at all stages, and RUE also showed a downward trend during the MA stage (Table 3). This phenomenon is associated with the significant reduction in LAI and Pn during the later stages of grain filling, leading to insufficient supply of photosynthetic products and a subsequent decline in biomass yield [33].

2-Acetyl-1-pyrroline (2-AP) is the key volatile compound responsible for the characteristic aroma of aromatic rice [1,38,39]. In this study, both concentrations of PBZ treatments increased the 2-AP content in grains at maturity, with the T1 treatment showing a more pronounced enhancement (Figure 1B). This is consistent with the findings of Xing et al. [12]. Concurrently, PBZ treatments also elevated the levels of proline and Δ1-pyrroline (Figure 3). As critical precursors for 2-AP synthesis, the increased content of proline and Δ1-pyrroline significantly promoted 2-AP accumulation [39]. The results of correlation analysis also confirmed this conclusion (Figure 4). Proline plays a pivotal role in plant stress responses, and its content variation is usually closely linked to plant stress resistance [40]. PBZ application may activate endogenous protective mechanisms by altering hormonal levels in plants, leading to increased proline synthesis and thereby promoting 2-AP formation [40,41].

The biosynthesis of 2-AP is not only regulated by genetic factors but also closely related to carbon and nitrogen metabolism as well as photosynthetic efficiency [42,43]. Isotope tracing reveals that the nitrogen source of 2-AP is derived from proline [44], and its acetyl group may be supplied from glucose metabolism [45]. Thus, adequate carbon and nitrogen sources are critical for aroma enhancement. We found that 2-AP content was significantly positively correlated with above-ground biomass of rice plant at MA, Pn at FH14, and RUE at FH and MA stages (Figure 4). This indicates that these traits, directly related to photosynthetic efficiency and light interception, play a key role in the biosynthesis of 2-AP. Previous studies have shown that moderate shading can increase the content of 2-AP in aromatic rice [11]. In this study, PBZ application improved 2-AP content, but significantly reduced LAI, in turn enhancing the canopy’s light transmittance. To further reveal the underlying patterns, we developed interaction models of 2-AP with IPAR and RUE based on different concentrations of PBZ treatment. Our data showed that under PBZ treatments (T1 and T2), the content of 2-AP exhibited a dependence on both IPAR and RUE (Figure 5). Low-level IPAR indicates stronger light transmittance within the canopy. However, excessively high-level IPAR will weaken the ability of the canopy to transform intercepted light energy into dry matter, thereby decreasing RUE. The enhancement of photosynthetic efficiency promotes the accumulation of photosynthetic products, thereby providing more carbon skeletons and energy for 2-AP biosynthesis [7,46]. Therefore, moderate-level IPAR and RUE contribute to high-level aroma maintenance.

The formation mechanisms of yield and aroma content in aromatic rice are intricately intertwined and influenced by a diverse array of factors. For instance, while moderate shading can enhance the content of 2-AP, it may concurrently reduce photosynthetic efficiency, thereby exerting a negative impact on yield [11,47]. Therefore, optimizing the cultivation environment and practices is crucial for improving the quality and yield of aromatic rice. In recent years, research on using growth regulators to boost the growth of aromatic rice and increase its aroma content has gradually increased [12,48,49]. Taken together, this study revealed that appropriate concentration PBZ application elevated Pn and enhanced accumulation and transport of dry matter, thus facilitating high yield and aroma formation of aromatic rice. The low cost of PBZ enables it to be used in large-scale rice production. Although the PBZ dose used in this study falls within the safety range reported in previous studies [50,51], long-term and high-frequency application may cause its entry into water bodies via surface runoff, thereby posing a potential threat to groundwater and aquatic organisms. Therefore, potential environmental risk should be evaluated prior to long-term PBZ application.

## 5. Conclusions

Our study indicated that foliar application of PBZ at around 150 mg L^−1^ at the panicle initiation stage not only enhanced rice yield but also increased the 2-AP content in grains of aromatic rice at maturity. The yield increase was significantly correlated with higher GNP and TGW derived from elevated net photosynthetic rates during grain filling, promoting dry matter accumulation and transport, above-ground biomass, and canopy radiation use efficiency under appropriate PBZ application. These effects also collectively provide more carbon skeletons and energy for 2-AP biosynthesis. On the other hand, PBZ application increased the content of key precursors for 2-AP synthesis (proline and Δ1-pyrroline), thereby intensifying aroma. This study provides an effective technical support for the production of high-yield aroma-enhanced rice by applying PBZ. Future studies are suggested to elucidate the underlying mechanisms by which PBZ regulates 2-AP biosynthesis in aromatic rice from the perspective of carbon and nitrogen metabolism.

## Figures and Tables

**Table 1:** Effect of foliar spraying of paclobutrazol on above-ground biomass of aromatic rice (g m^−2^).

Year	Cultivar	Treatment	FH	FH14	MA
2022	MXZ2	CK	677.93 ± 47.13 a	925.42 ± 38.17 b	1196.81 ± 17.06 a
		T1	684.07 ± 29.52 a	1045.21 ± 41.33 a	1268.61 ± 55.97 a
		T2	698.69 ± 38.39 a	1005.92 ± 38.86 a	1167.87 ± 70.35 a
	XYXZ	CK	693.03 ± 20.32 a	909.67 ± 41.52 b	1290.58 ± 29.44 ab
		T1	715.85 ± 37.20 a	987.71 ± 31.71 a	1359.71 ± 46.22 a
		T2	700.04 ± 35.76 a	940.46 ± 34.94 ab	1234.43 ± 45.43 b
2023	MXZ2	CK	709.66 ± 43.30 a	877.71 ± 46.88 a	1252.51 ± 20.70 b
		T1	703.60 ± 16.87 a	932.46 ± 39.14 a	1316.78 ± 13.40 a
		T2	742.60 ± 29.63 a	916.92 ± 24.80 a	1200.43 ± 42.40 b
	XYXZ	CK	695.42 ± 37.93 a	879.79 ± 21.56 b	1237.15 ± 54.27 ab
		T1	718.94 ± 50.58 a	928.75 ± 16.24 ab	1306.65 ± 37.62 a
		T2	735.93 ± 22.90 a	988.21 ± 45.81 a	1190.02 ± 64.62 b
Analysis of variance (ANOVA)					
Year			n.s.	*	n.s.
Cultivar			n.s.	n.s.	n.s.
Treatment			n.s.	**	***
Year × Cultivar			n.s.	**	**
Year × Treatment			n.s.	n.s.	n.s.
Cultivar × Treatment			n.s.	n.s.	n.s.
Year × Cultivar × Treatment			n.s.	n.s.	n.s.

Note: CK, water control; T1, 150 mg L^−1^ PBZ; T2, 300 mg L^−1^ PBZ; FH, full heading stage; FH14, 14 days after heading; MA, maturity stage. MXZ2, Meixiangzhan 2; XYXZ, Xiangyaxiangzhan. Different lowercase letters indicate a significant difference at 5% probability level among treatments of the same rice cultivar in the same year. *, *p* < 0.05; **, *p* < 0.01; ***, *p* < 0.001; n.s., not significant (*p* > 0.05).

**Table 2:** The effect of foliar spraying of paclobutrazol on the yield composition.

Year	Cultivar	Treatment	EPN (m^2^)	GNP	SSR (%)	TGW (g)
2022	MXZ2	CK	273.49 ± 8.64 b	137.72 ± 4.69 ab	76.21 ± 1.56 b	19.29 ± 0.51 ab
		T1	284.69 ± 15.44 b	143.72 ± 7.67 a	84.51 ± 1.16 a	20.87 ± 1.20 a
		T2	307.70 ± 13.90 a	126.90 ± 9.82 b	75.09 ± 2.49 b	17.91 ± 0.47 b
	XYXZ	CK	254.93 ± 8.52 a	121.37 ± 5.75 b	73.27 ± 2.67 b	18.15 ± 0.26 b
		T1	267.60 ± 11.27 a	133.11 ± 6.03 a	81.55 ± 3.59 a	19.59 ± 0.49 a
		T2	255.41 ± 12.44 a	122.21 ± 2.91 b	75.37 ± 3.13 ab	16.93 ± 0.61 c
2023	MXZ2	CK	284.99 ± 21.85 a	145.37 ± 7.80 ab	75.00 ± 1.27 b	19.01 ± 0.57 a
		T1	304.99 ± 18.62 a	153.85 ± 6.36 a	83.32 ± 2.65 a	19.98 ± 0.55 a
		T2	286.14 ± 25.34 a	133.33 ± 10.14 b	80.19 ± 2.83 a	17.59 ± 0.51 b
	XYXZ	CK	246.96 ± 13.82 a	127.83 ± 6.88 b	77.45 ± 2.52 b	18.64 ± 0.44 a
		T1	276.18 ± 19.86 a	142.07 ± 3.39 a	84.03 ± 1.91 a	19.50 ± 0.44 a
		T2	267.72 ± 15.27 a	114.60 ± 6.10 c	71.53 ± 2.19 c	17.58 ± 0.52 b
Analysis of variance (ANOVA)						
Year			n.s.	n.s.	n.s.	n.s.
Cultivar			***	***	n.s.	n.s.
Treatment			n.s.	**	***	***
Year × Cultivar			n.s.	n.s.	n.s.	*
Year × Treatment			n.s.	n.s.	n.s.	n.s.
Cultivar × Treatment			n.s.	n.s.	n.s.	*
Year × Cultivar × Treatment			*	n.s.	**	n.s.

Note: EPN, effective panicle number; GNP, grain number per panicle; SSR, seed setting rate; TGW, 1000-grain weight; CK, water control; T1, 150 mg L^−1^ PBZ; T2, 300 mg L^−1^ PBZ; MXZ2, Meixiangzhan 2; XYXZ, Xiangyaxiangzhan. Different lowercase letters indicate a significant difference at 5% probability level among treatments of the same rice cultivar in the same year. *, *p* < 0.05; **, *p* < 0.01; ***, *p* < 0.001; n.s., not significant (*p* > 0.05).

**Table 3:** Effect of paclobutrazol on the radiation utilization rate of aromatic rice.

Year	Cultivar	Treatment	IPAR (MJ m^−2^)			RUE (g MJ^−1^)		
			FH	FH14	MA	FH	FH14	MA
2022	MXZ2	CK	522.47 ± 11.56 a	568.10 ± 8.51 a	341.44 ± 15.8 a	1.07 ± 0.10 b	0.44 ± 0.08 b	0.80 ± 0.10 a
		T1	473.18 ± 8.59 b	451.84 ± 10.72 b	294.66 ± 10.25 b	1.20 ± 0.04 ab	0.80 ± 0.07 a	0.76 ± 0.06 a
		T2	433.99 ± 10.50 c	408.04 ± 3.88 c	239.60 ± 22.25 c	1.33 ± 0.11 a	0.75 ± 0.10 a	0.67 ± 0.10 a
	XYXZ	CK	514.55 ± 13.24 a	561.09 ± 6.07 a	298.17 ± 6.24 a	1.13 ± 0.05 b	0.39 ± 0.07 b	1.28 ± 0.10 b
		T1	480.87 ± 13.06 b	528.29 ± 2.54 b	218.99 ± 7.87 b	1.25 ± 0.13 b	0.52 ± 0.07 b	1.70 ± 0.03 a
		T2	353.63 ± 7.77 c	329.63 ± 11.17 c	189.62 ± 9.81 c	1.35 ± 0.08 a	0.73 ± 0.08 a	1.55 ± 0.09 a
2023	MXZ2	CK	547.49 ± 13.91 a	548.03 ± 13.19 a	318.37 ± 15.73 a	1.08 ± 0.10 b	0.31 ± 0.01 c	1.18 ± 0.07 a
		T1	509.05 ± 10.74 b	520.57 ± 40.48 ab	320.58 ± 5.97 a	1.15 ± 0.02 b	0.44 ± 0.03 a	1.20 ± 0.11 a
		T2	419.66 ± 10.22 c	467.82 ± 19.31 b	271.50 ± 4.20 b	1.30 ± 0.08 a	0.37 ± 0.02 b	1.04 ± 0.08 a
	XYXZ	CK	513.91 ± 10.53 a	510.95 ± 21.80 a	299.83 ± 23.72 a	1.13 ± 0.03 b	0.36 ± 0.02 b	1.19 ± 0.02 b
		T1	415.83 ± 10.33 b	504.58 ± 24.26 a	254.97 ± 14.49 b	1.44 ± 0.13 a	0.41 ± 0.06 ab	1.48 ± 0.06 a
		T2	404.10 ± 7.87 b	469.99 ± 17.78 a	206.48 ± 24.94 c	1.55 ± 0.04 a	0.54 ± 0.12 a	0.98 ± 0.08 c
Analysis of variance (ANOVA)								
Year			n.s.	n.s.	n.s.	n.s.	***	n.s.
Cultivar			n.s.	n.s.	***	*	n.s.	***
Treatment			***	***	***	***	**	n.s.
Year × Cultivar			**	n.s.	n.s.	n.s.	***	***
Year × Treatment			**	***	**	n.s.	**	**
Cultivar × Treatment			*	***	*	n.s.	**	***
Year × Cultivar × Treatment			***	***	n.s.	**	n.s.	**

Note: IPAR, intercepted photosynthetically active radiation; RUE, radiation use efficiency; CK, water control; T1, 150 mg L^−1^ PBZ; T2, 300 mg L^−1^ PBZ; FH, full heading stage; FH14, 14 days after heading; MA, maturity stage. MXZ2, Meixiangzhan 2; XYXZ, Xiangyaxiangzhan. Different lowercase letters indicate a significant difference at 5% probability level among treatments of the same rice cultivar in the same year. *, *p* < 0.05; **, *p* < 0.01; ***, *p* < 0.001; n.s., not significant (*p* > 0.05).

## Data Availability

Data will be made available on request.
